# Assessing oral and dental health-related quality of life in head and neck cancer patients at 1, 5, and 10 years: a comparison of the EORTC QLQ43, FACT H&N, and the Orthognathic-QLQ questionnaires

**DOI:** 10.1007/s12094-025-03895-0

**Published:** 2025-04-10

**Authors:** Marc Guedea, Meritxell Sánchez, Alicia Lozano, Montse Ferrer, Angels Pont, Sandra Clotet, Marc Juárez, Isabel Linares, Pablo Araguas, Montse Ventura, Nuno Gustavo d’Oliveira, Maria Cristina Manzanares, Josep Maria Ustrell, Ferran Guedea

**Affiliations:** 1 Orthodontics Department. Facultat de Medicina I Ciències de la Salut, University of Barcelona (UB), Barcelona, Spain; 2 Radiation Oncology Department, Institut Català d’Oncologia (ICO), Hospital Duran I Reynals, L’Hospitalet de Llobregat, Barcelona, Spain; 3 Head and Neck Cancer Tumor Board, Institut Català d’Oncologia (ICO), Hospital Duran I Reynals, L’Hospitalet de Llobregat, Barcelona, Spain; 4 Health Services Research Group, Hospital del Mar Research Institut, IMIM, Barcelona, Spain; 5 CIBER Epidemiología y Salud Pública (CIBERESP), Universitat Pompeu Fabra, Barcelona, Spain; 6 Clinical Sciences Departament, Facultat de Medicina i Ciències de la Salut, University of Barcelona (UB), L’Hospitalet de Llobregat, 08098 Barcelona, Spain; 7 University of Barcelona (UB), Barcelona, Spain

**Keywords:** Health-related quality of life, EORTC QLQ-H&N43, FACT H&N, Orthognathic-QLQ, Dental care, Head and neck cancer, Radiotherapy

## Abstract

**Purpose:**

Numerous health-related quality of life (HRQoL) questionnaires are available to assess oral HRQoL in patients undergoing treatment for head and neck (H&N) cancer. These multidimensional instruments should have the capacity to detect meaningful clinical oral function/dental changes at different time points. However, the optimal instrument—or combination of instruments—for assessing oral health and dental needs is not clear. We administered three questionnaires (FACT H&N, EORTC QLQ-H&N43, Orthognathic-QLQ) to assess oral and dental HRQoL in a cohort of H&N cancer patients at 1-, 5- and 10-year post-treatment. A secondary aim was to compare these questionnaires to determine which provides the most useful assessment of oral HRQoL and dental care needs.

**Methods:**

Prospective, single-center study of patients (*n* = 82) with H&N cancer grouped according to the follow-up time (1, 5, or 10 years). HRQoL was assessed by telephone with the Functional Assessment of Cancer Therapy (FACT H&N), the European Organization for Research and Treatment (EORTC QLQ-H&N43), and the Orthognathic Questionnaire (OQLQ). Analyses were performed to assess differences between groups.

**Results:**

Eighty-two patients (fifty-nine men) were included. The mean age was 61.9 years. On the EORTC QLQ-H&N43, significant between-group differences (1 year vs. 5 and 10 years) were observed on five multi-item scales (mouth pain, senses, body image, anxiety, and shoulder problems) and on three single-item scales (neurological problems, neck swelling, and weight loss), indicating that QoL for those domains was more negatively impacted at 1-year post-treatment. Adjusted mean scores on most items on the EORTC QLQ-H&N43 were similar in the 5- and 10-year groups. On the other two scales (FACT H&N and OQLG), there were no significant between-group (1, 5, 10 years) differences in adjusted mean scores.

**Conclusion:**

These results show that the negative impact of H&N cancer on HRQoL is most evident at 1-year versus 5- or 10-year post-treatment. The combined administration of the EORTC QLQ-H&N43 and the OQLQ appear to provide the most useful assessment of HRQoL.

## Introduction

Head and neck (H&N) cancer comprises malignant epithelial tumors originating in the oral cavity, paranasal sinuses, nasal cavity, pharynx, and larynx [1]. Globally, H&N cancer is the seventh most common cancer, with around 660,000 new cases and 325,000 deaths each year [2]. H&N cancer accounts for 5% of all adult cancer cases in Spain [3]. Known risk factors include tobacco use and alcohol consumption. Human papillomavirus (HPV) infection has been recognized as a risk factor for H&N cancer, mainly oropharyngeal cancer [4]. While the global incidence of H&N cancer has slowly declined in recent decades, partly due to reduced smoking rates, HPV-related oropharyngeal cancer cases continue to rise [4]. In most cases, the treatment of H&N cancer requires a multimodal approach involving surgery, radiation therapy, and chemotherapy managed by a multidisciplinary team. Standard radiation therapy consists of 60 to 70 Gy administered over a 5-day week. Cisplatin/carboplatin or cetuximab are often used as radiosensitizers. Recently, targeted immunotherapy has been introduced for select patients with H&N tumors [5].

In many cancers, including H&N cancer, treatment advances have extended life expectancy. As a result, health-related quality of life (HRQoL) has become an increasingly important outcome measure for long-term survivors. The two main tools used to evaluate HRQoL in patients with H&N cancer are the Functional Assessment of Cancer Therapy in Head and Neck (FACT H&N) [6] and the European Organization for Research and Treatment of Cancer Quality of Life-Head and Neck Cancer (EORTC QLQ-H&N43) [7, 8]. Due to their anatomical location, these cancers can have a significant negative impact on oral and dental health. Although both the FACT H&N and the EORTC QLQ-H&N43 assess some aspects of oral health, neither of these instruments was designed specifically to assess dental health. By contrast, the Orthognathic Quality of Life Questionnaire (Orthognathic-QLQ) [9, 10] is a more targeted instrument developed to assess overall dental QoL, including facial esthetics, oral function, and awareness of dental deformity.

Although a growing number of survivors seek dental care to maintain optimal dental function and esthetics, the impact of H&N cancer on dental-related QoL remains underexplored. Moreover, it is unclear whether the currently available HRQoL instruments provide an adequate assessment of oral HRQoL in patients with H&N cancer.

In this context, the main aim of the present cross-sectional study was to assess and compare oral and dental HRQoL in a cohort of H&N cancer patients at 1, 5, and 10 years after treatment completion. A second aim was to determine which of these three questionnaires (FACT H&N, EORTC QLQ-H&N43, Orthognathic-QLQ) provide the most useful assessment of oral HRQoL and the need for dental care.

## Materials and methods

### Design and participants

This was a prospective study conducted in 2023 and 2024 involving 82 H&N cancer patients who were evaluated at 1 year (*n* = 28 patients), 5 years (*n* = 33) and 10 years (*n* = 21) following successful treatment for H&N cancer. All patients were diagnosed and treated for H&N cancer at the Institut Català d’Oncologia (ICO) in L’Hospitalet de Llobregat, Barcelona (Spain). The ICO is a public, specialized cancer center that serves over 50% of the adult population in Catalonia.

The inclusion criteria for the study were as follows: age ≥ 18 years; diagnosis of H&N cancer (oral cavity, hypopharynx, oropharynx, nasopharynx, and/or larynx); and treatment with radical radiotherapy with or without chemotherapy. Surgically treated patients were excluded. Patients with primary laryngeal tumors, neoplasms in other parts of the body, and those who did not receive radiotherapy as their main cancer treatment were excluded.

The study was approved by the Clinical Research Ethics Committee (CEIC) of the ICO and the Hospital Universitari de Bellvitge (approval code: PR354/22; approval date: February 9, 2023), L’Hospitalet de Llobregat, Barcelona, Spain. All patients provided signed written informed consent.

### Study procedures

Patients participated in a computer-assisted telephone interview conducted by a trained, experienced interviewer who administered the three study questionnaires. Two of the instruments assess HRQoL in H&N cancer (FACT H&N Symptom Index and the EORTC QLQ-H&N43). The third instrument (Orthognathic-QLQ) was developed to assess oral HRQoL in patients with severe dentofacial deformity.

The FACT H&N is a 39-item HRQoL questionnaire designed for H&N cancer patients. It comprises two modules, the FACT-General and the Head and Neck Cancer module. The FACT Head & Neck Symptom Index, derived from the FACT H&N, focuses on the ten items most relevant to disease symptoms [6]. This questionnaire uses a 7-day response period and a 5-point Likert-type scale ranging from 1 "not at all" to 5 “very much”.

The EORTC QLQ-H&N43 is an updated version of the EORTC QLQ-H&N35 [7, 8]. This instrument has 12 multi-item scales to assess the following symptoms: dry mouth/sticky saliva; pain in the mouth; senses; social eating; swallowing; sexuality; body image; speech problems; teeth issues; anxiety; shoulder problems; and skin problems. It also has seven single-item symptom scales: coughing; opening mouth; social contact; neurological issues; neck swelling; weight loss; and wound healing problems. All of the items are rated on a 5-point Likert scale ranging from “not at all” to “very much”. [8].

For the purposes of the present study, the raw scores on the FACT H&N Symptom Index and the EORTC QLQ-H&N43 were converted to a 0 to 100 scale to facilitate comparisons between the questionnaires. Higher scores indicate worse HRQoL.

The Orthognathic-QLQ is a 22-item self-administered questionnaire covering 4 domains of oral HRQoL (facial esthetics, oral function, awareness of dental deformity, and overall dental QoL) rated on a 5-point Likert scale. Total scores by domain are as follows: facial esthetics (0–20 points), oral function (0–20), awareness of dental deformity (0–16), and overall dental QoL (0–32) [9, 10]. For the full scale, the total point range is 0 to 88. Higher scores indicate worse oral HRQoL. The scale has been shown to have good internal consistency, test–retest reliability, and patient acceptance of the questionnaire [9].

In addition to the three questionnaires, we collected data on the following variables: demographics (age and sex); smoking habit; alcohol use; tumor location; histological grade; tumor and lymph node stage; total radiation dose; surgical treatment (yes/no); and chemotherapy regimen (including cisplatin cycles).

### Statistical analysis

Categorical variables are reported as absolute numbers with percentages. Continuous variables are presented as means with standard deviation (SD). Bivariate analyses of the FACT H&N, EORTC QLQ-H&N43, and Orthognathic-QLQ were performed at both the dimension and item levels. At the item level, the Likert scales were dichotomized into two categories: “not at all” and all other responses. The response percentages for each group were compared using the Chi-square test. Mean scores for each group on the dimensions were compared using one-way analysis of variance (ANOVA). Post hoc comparisons to test differences between pairs of groups were performed with Dunnett’s T3 (assuming non-equal variances).

Multivariate analyses were performed with lineal regression models to assess differences between groups, with the 1-year group considered the reference category. Multivariate analyses were also performed among groups (type III for ANOVA) after adjusting for age and sex, and the clinical variables that presented statistically significant differences on the bivariate analyses. R (version 4.2.2), and RStudio (2022.07.2 Build 576) were used for all analyses. Statistical significance was set at p < 0.05.

## Results

### Patient characteristics

A total of 82 patients (59 men; 72%) were included in the study. The clinical characteristics of the participants are shown in Table 1. The mean age was 61.9 years. The most common tumor sites were as follows: oropharynx (*n* = 27, 34%), oral cavity (*n* = 17, 21%), nasopharynx (*n* = 16, 20%), and larynx (*n* = x, 17%). The most common disease stages in the 5- and 10-year groups were stage T1 and T3 disease. By contrast, stage T2 was more common in the 1-year group (Table 1).

**Table 1:** Clinical characteristics of the study population

Variables	Time after completing oncological treatment			*p* value
	1 year (*n* = 28)	5 years (*n* = 33)	10 years (*n* = 21)	
Sex, *n* (%)				
Men	19 (67.9%)	25 (75.8%)	15 (71.4%)	0.790
Women	9 (32.1%)	8 (24.2%)	6 (28.6%)	
Mean age (SD), years	60.8 (7.9)	60.0 (10.7)	66.2 (9.2)	0.052
Smoking habit, *n* (%)				
Current smoker	9 (32.1%)	11 (33.3%)	9 (42.9%)	0.784
Ex-smoker	13 (46.4%)	13 (39.4%)	6 (28.6%)	
Never smoker	6 (21.4%)	9 (27.3%)	6 (28.6%)	
Alcohol consumption, *n* (%)				
Never	16 (57.1%)	22 (66.7%)	13 (61.9%)	0.821 *
Occasional	7 (25.0%)	6 (18.2%)	3 (14.3%)	
Heavy	2 (7.1%)	4 (12.1%)	3 (14.3%)	
Missing	3 (10.7%)	1 (3.0%)	2 (9.5%)	
Primary cancer site, *n* (%)				
Hypopharynx	2 (7.1%)	2 (6.1%)	3 (14.3%)	0.354
Nasopharynx	4 (14.3%)	7 (21.2%)	5 (23.8%)	
Larynx	3 (10.7%)	9 (27.3%)	2 (9.5%)	
Oral cavity	8 (28.6%)	7 (21.2%)	2 (9.5%)	
Oropharynx	11 (39.3%)	8 (24.2%)	9 (42.9%)	
Tumor grade, *n* (%)				
Grade 1	2 (7.1%)	1 (3.0%)	0 (0.0%)	0.340 *
Grade 2	3 (10.7%)	7 (21.2%)	1 (4.8%)	
Grade 3	2 (7.1%)	0 (0.0%)	0 (0.0%)	
Unknown	21 (75.0%)	25 (75.8%)	20 (95.2%)	
Tumor stage, *n* (%)				
T1	5 (17.9%)	12 (36.4%)	7 (33.3%)	0.008 *
T2	8 (28.6%)	5 (15.2%)	5 (23.8%)	
T3	7 (25.0%)	12 (36.4%)	6 (28.6%)	
T4	5 (17.9%)	3 (9.1%)	3 (14.3%)	
Unknown	3 (10.7%)	1 (3.0%)	0 (0.0%)	
Nodal stage, *n* (%)				
N0	12 (42.9%)	11 (33.3%)	5 (23.8%)	0.007 *
N1	4 (14.3%)	7 (21.2%)	3 (14.3%)	
N2	8 (28.6%)	14 (42.4%)	13 (61.9%)	
N3	2 (7.1%)	0 (0.0%)	0 (0.0%)	
Unknown	2 (7.1%)	1 (3.0%)	0 (0.0%)	
Radiation dose, Gy, mean (SD)	67.1 (4.4)	67.5 (5.3)	68.2 (4.8)	0.052
Surgical excision of tumor, *n* (%)				
No	17 (60.7%)	24 (72.7%)	18 (85.7%)	0.155
Yes	11 (39.3%)	9 (27.3%)	3 (14.3%)	
Chemotherapy, *n* (%)				
Yes	15 (55.6%)	23 (69.7%)	20 (95.2%)	0.006
No	13 (46.4%)	10 (30.3%)	1 (4.8%)	
Cisplatin				
Yes	10 (58.8%)	17 (68.0%)	16 (84.2%)	0.234
No	7 (41.2%)	8 (32.0%)	3 (15.8%)	
Cycles of cisplatin, mean (SD)	3.2 (1.9)	2.3 (0.6)	2.7 (0.5)	
Use of cetuximab	2 (7.1%)	4 (12.1%)	3 (14.3%)	
Cycles of cetuximab, mean (SD)	6.5 (0.7)	6.3 (1.5)	6.3 (2.9)	

SD: standard deviation

*p value excluding unknown patients

All participants received 33 sessions of radical radiotherapy. In all patients, the radiotherapy technique was volumetric modulated arc therapy (VMAT), performed once daily, 5 days a week for 7 weeks. The total dose to the tumor ranged from 60 to 70 Gy (2 to 2.12 Gy per fraction) and 54.12 Gy to the lymph node areas at risk of subclinical disease (1.64 Gy per fraction). Most of the patients (70.7%) underwent chemotherapy (mainly cisplatin-based), which was delivered concurrently with radiotherapy. No significant differences were observed in baseline clinical variables among the three groups, except for tumor stage (*p* = 0.008), nodal stage (N0: 42.9%, 33.3% and 23.8%, respectively; *p* = 0.007), and chemotherapy (55.6%, 69.7%, and 95.2%, respectively, *p* = 0.006).

### Quality of life assessed with the FACT H&N symptom index

**Fig. 1:**
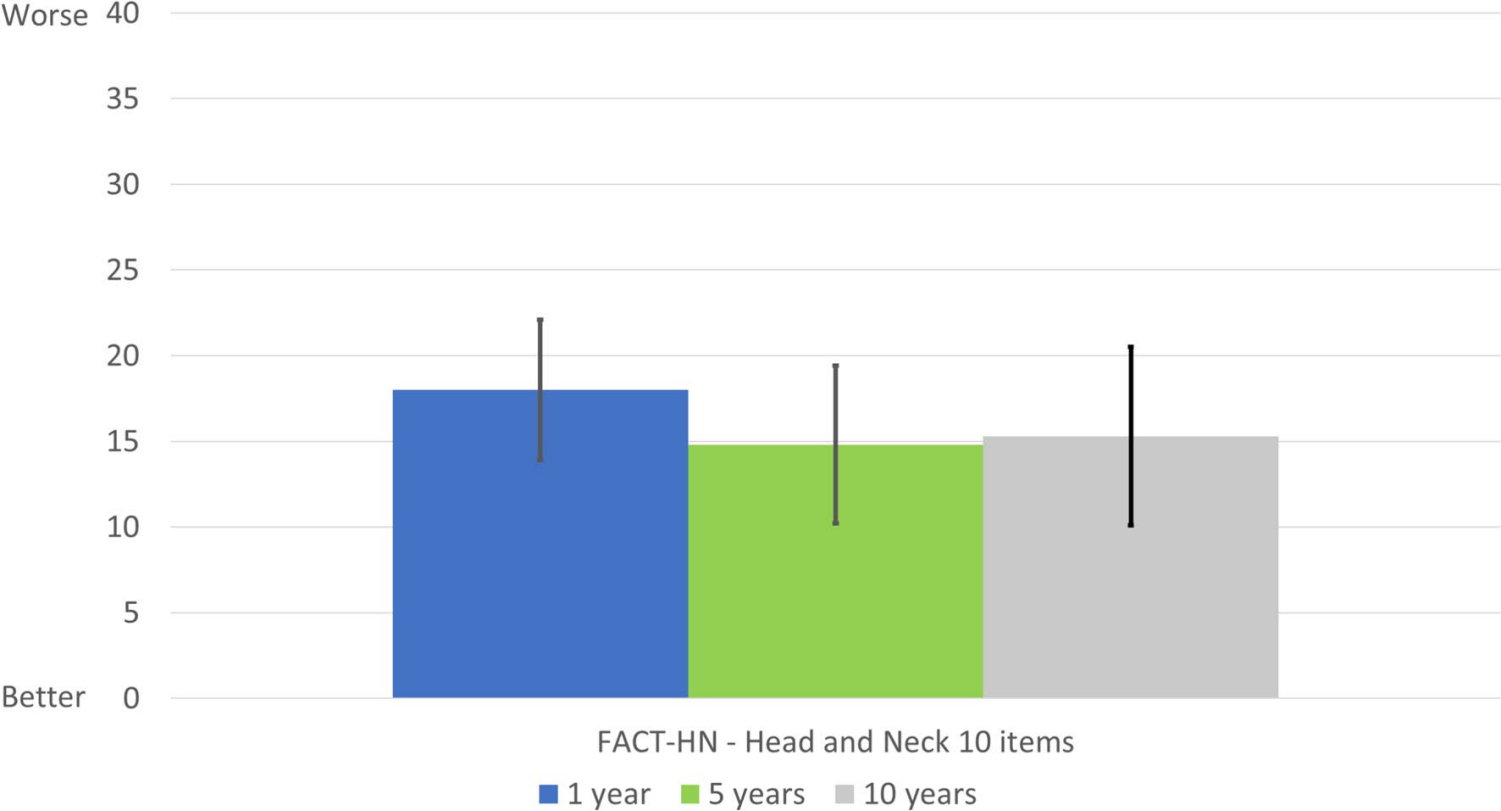
FACT H&N Symptom Index total score on a scale of 0 to 100 (worst HRQoL): adjusted means with 95% confidence intervals by group (1, 5, or 10 years after treatment completion)

Figure 1 shows the adjusted mean scores with 95% confidence intervals (CI) for the total FACT H&N Symptom Index. Table 2 shows the crude and adjusted mean total scores. As that table shows, there were no significant differences between the groups (*p* = 0.277 and *p* = 0.883, respectively).

**Table 2:** FACT H&N Symptom Index total score and percentage of patients reporting symptoms at 1-, 5- and 10-year post-treatment

Variables	Time after completing oncological treatment			*p* value
	1 year (*n* = 28)	5 years (*n* = 33)	10 years (*n* = 21)	
Total score, mean (SD)	16.6 (9.0)	13.5 (6.5)	15.0 (6.9)	0.277
Total score, adjusted mean {SE}	18.0 {2.0}	14.8 {2.3}	15.3 {2.6}	0.883
Symptom, *n* (%)*				
I have pain	22 (78.6%)	25 (75.8%)	20 (95.2%)	0.170
I have a lack of energy	16 (57.1%)	18 (54.5%)	13 (61.9%)	0.867
I can swallow naturally and easily	17 (60.7%)	23 (69.7%)	18 (85.7%)	0.161
I have pain in my mouth, throat or neck	16 (57.1%)	15 (45.5%)	8 (38.1%)	0.398
I have trouble breathing	8 (28.6%)	9 (27.3%)	7 (33.3%)	0.888
I am able to communicate with others	17 (60.7%)	23 (69.7%)	10 (47.6%)	0.268
I have nausea	7 (25.0%)	3 (9.1%)	1 (4.8%)	0.077
I can eat solid foods	20 (71.4%)	24 (72.7%)	16 (76.2%)	0.830
I worry that may condition will get worse	27 (96.4%)	31 (93.9%)	20 (95.2%)	0.903
I am content with the quality of my life now	18 (64.3%)	14 (42.4%)	14 (66.7%)	0.121

*Number of patients reporting all response options except for “not at all”

Post hoc comparisons with *p* < 0.05: **a** Between 1- and 5-year post-treatment, **b** Between 1- and 10-year post-treatment, **c** Between 5- and 10-year post-treatment

The most common positive responses to the FACT H&N in the 1-year group were observed for the following items: “*I worry that my condition will get worse”* (96.4% of respondents) and “*I can swallow naturally and easily”* (89.3%). In the 5-year group, the item with the highest affirmative response rate was “*I am content with the quality of my life right now”* (100%). In the 10-year group, more than 95% of patients responded affirmatively to the items “*I have pain”* (95.2%) and “*I worry that my condition will get worse”* (95.2%).

### Quality of life assessed with the EORTC QLQ-H&N43 questionnaire

**Fig. 2:**
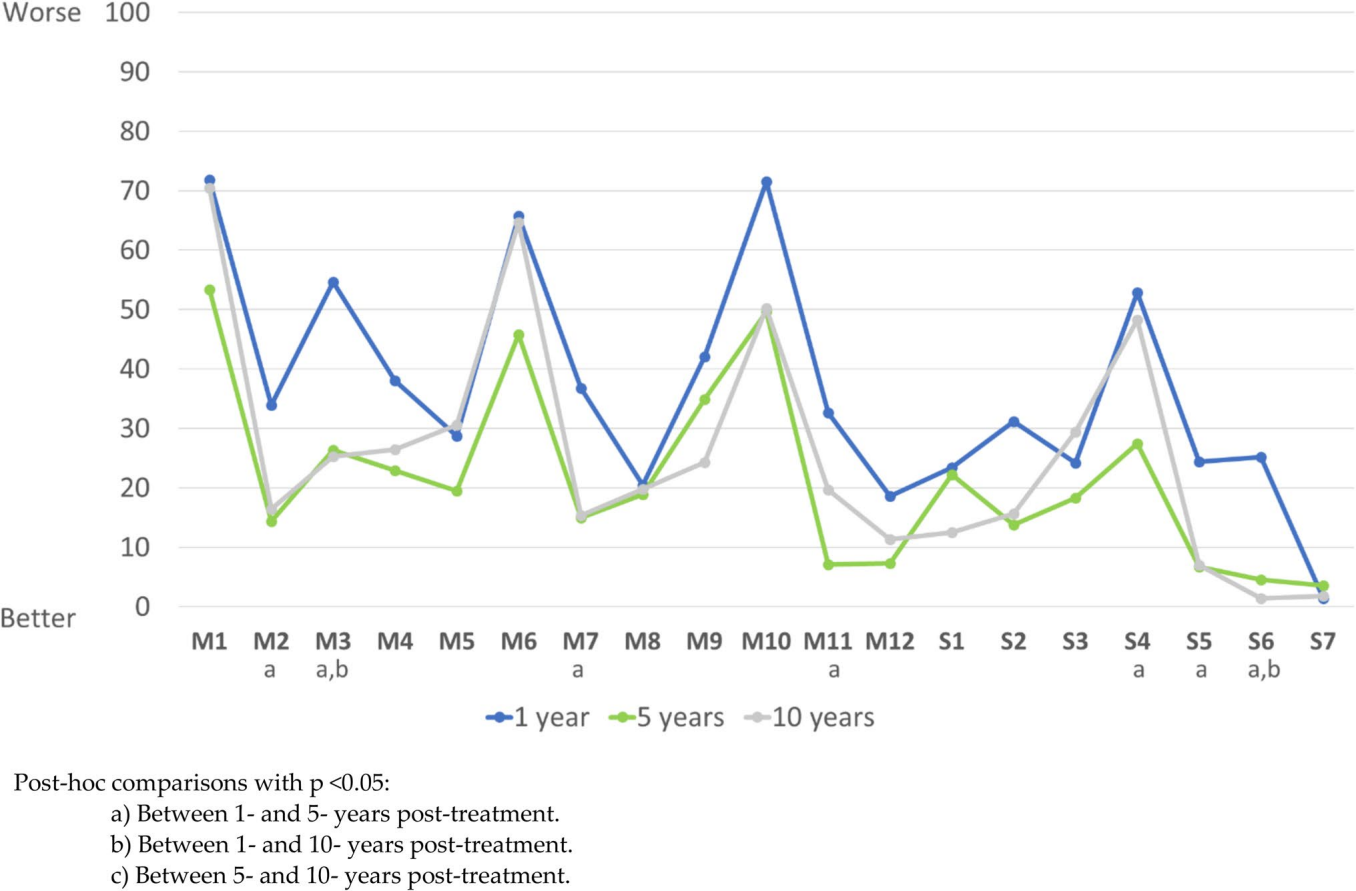
EORTC QLQ-H&N43 scores of the 12 multi-item scales and the 7 single-item symptom scales, from 0 to 100 (worst HRQoL): adjusted means in patients evaluated after 1, 5, and 10 years of completing treatment for head and neck cancer

Almost all domains of the EORTC QLQ-H&N questionnaire showed better HRQoL after 5 years of treatment than after 1 year (Fig. 2). However, differences between the adjusted means were only statistically significant for four of the multi-item scales and three of the single-item scales. The adjusted mean scores on most dimensions were similar (no statistically significant differences) in the 5-year and 10-year groups. Five scales (M1, M5, M6, S3 and S4) were similar to the 1-year group.

Table 3 shows the crude and adjusted mean scores on the multi-item scales and the single-item symptom scales for all three groups. HRQoL was significantly worse (adjusted mean scores) in the 1-year vs. the 5-year group for the following multi-item domains: pain in the mouth (M2, *p* = 0.017); problems with senses (M3, *p* < 0.001); body image (M7, *p* = 0.001); anxiety (M10, *p* = 0.004), shoulder problems (M11, *p* = 0.002); neurological problems (S4, *p* = 0.005); swelling in the neck (S5, *p* = 0.003); and weight loss (S6, *p* < 0.001). The 1-year and 10-year groups showed significant differences in adjusted mean scores on the M3, M10, and S6. There were no significant differences between the 5- and 10-year groups on any scale. The differences observed on the bivariate analysis for M1 (dry mouth/sticky saliva), M5 (swallowing), and M6 (sexuality) were no longer present after adjusting for age, sex, tumor stage, and nodal stage.

**Table 3:** EORTC QLQ-H&N43 scores at 1, 5, and 10 years after treatment completion

Variables	Time after completing oncological treatment			*p* value
	1 year (*n* = 28)	5 years (*n* = 33)	10 years (*n* = 21)	
Multi-item scales				
M1: dry mouth/sticky saliva crude mean score (SD) Adjusted mean score {SE}	61.9 (34.2) 71.8 {8.6}	50.0 (35.4) 53.4 {9.7}	74.6 (28.7) 70.5 {10.8}	0.034 c 0.083
Have you had a dry mouth?, *N* (%) *	25 (89.3%)	27 (81.8%)	20 (95.2%)	0.325
Have you had sticky saliva?, *N* (%) *	18 (64.3%)	17 (53.1%)	18 (85.7%)	0.050
M2: pain in the mouth crude mean score (SD) Adjusted mean score {SE}	29.5 (31.5) 33.9 {6.6}	9.6 (14.5) 14.4 {7.5}	13.9 (19.8) 16.4 {8.4}	0.004 a 0.017 a
Have you had pain in your mouth?, *N* (%) *	15 (53.6%)	4 (12.1%)	4 (19.0%)	0.001
Have you had pain in your jaw?, *N* (%) *	13 (46.4%)	4 (12.1%)	5 (23.8%)	0.010
Have you had soreness in your mouth?, *N* (%) *	18 (64.3%)	10 (30.3%)	7 (33.3%)	0.017
Have you had pain in your throat?, *N* (%) *	10 (35.7%)	12 (36.4%)	9 (42.9%)	0.857
M3: problems with senses crude mean score (SD) Adjusted mean score {SE}	50.0 (34.5) 54.6 {9.3}	24.7 (28.9) 26.4 {10.5}	23.8 (36.4) 25.3 {11.7}	0.005 a b < 0.001 a b
Have you had problems with your sense of smell?, *N* (%) *	14 (50.0%)	12 (36.4%)	7 (33.3%)	0.421
Have you had problems with your sense of taste?, *N* (%) *	22 (78.6%)	19 (57.6%)	8 (38.1%)	0.016
M4: social eating crude mean score (SD) Adjusted mean score {SE}	36.3 (38.0) 38.0 {7.5}	18.7 (21.4) 22.9 {8.5}	27.4 (28.8) 26.5 {9.5}	0.077 0.425
Have you had problems eating?, *N* (%) *	17 (60.7%)	20 (60.6%)	16 (76.2%)	0.438
Have you had problems eating in front of family?, *N* (%) *	9 (32.1%)	1 (3.0%)	3 (14.3%)	0.008
Have you had problems eating in front of other people?, *N* (%) *	12 (42.9%)	9 (27.3%)	7 (33.3%)	0.439
Have you had problems enjoying your meals?, *N* (%) *	14 (50.0%)	13 (39.4%)	7 (33.3%)	0.479
M5: swallowing crude mean score (SD) Adjusted mean score {SE}	26.2 (23.5) 28.7 {5.3}	17.7 (15.0) 19.5 {6.0}	32.5 (21.4) 30.6 {6.7}	0.028 c 0.080
Have you had problems swallowing liquids?, *N* (%) *	3 (10.7%)	2 (6.1%)	3 (14.3%)	0.597
Have you had problems swallowing pureed food?, *N* (%) *	6 (21.4%)	2 (6.1%)	4 (19.0%)	0.192
Have you had problems swallowing solid food?, *N* (%) *	21 (75.0%)	23 (69.7%)	18 (85.7%)	0.408
Have you choked when swallowing?, *N* (%) *	16 (57.1%)	17 (51.5%)	16 (76.2%)	0.185
M6: sexuality crude mean score (SD) Adjusted mean score {SE}	52.4 (43.0) 65.7 {11.7}	25.3 (36.4) 45.8 {13.2}	51.6 (47.4) 64.7 {14.7}	0.021 a 0.227
Have you felt less interest in sex? *N* (%) *	18 (64.3%)	11 (33.3%)	12 (57.1%)	0.041
Have you felt less sexual enjoyment? *N* (%) *	17 (60.7%)	11 (33.3%)	11 (55.0%)	0.080
M7: body image crude mean score (SD) Adjusted mean score {SE}	36.5 (41.9) 36.8 {7.9}	9.8 (19.8) 15.0 {8.9}	14.3 (31.8) 15.4 {9.9}	0.004 a 0.001 a
Have you had problems with your appearance?, *N* (%) *	13 (46.4%)	10 (30.3%)	5 (23.8%)	0.213
Have you felt less physically attractive as a result of your disease or treatment?, *N* (%)*	13 (46.4%)	7 (21.2%)	3 (14.3%)	0.024
Have you felt dissatisfied with your body?, *N* (%) *	12 (42.9%)	4 (12.1%)	4 (19.0%)	0.017
M8: speech problems crude mean score (SD) Adjusted mean score {SE}	24.3 (26.2) 20.5 {6.8}	24.8 (23.8) 18.9 {7.7}	24.1 (27.2) 19.8 {8.6}	0.994 0.094
Have you had problems with hoarseness?, *N* (%) *	15 (53.6%)	24 (72.7%)	13 (61.9%)	0.298
Have you had problems taking to other people?	7 (25.0%)	10 (30.3%)	5 (23.8%)	0.840
Have you had problems taking on the telephone?, *N* (%) *	6 (21.4%)	7 (21.2%)	5 (23.8%)	0.972
Have you had problems in a noisy environment?, *N* (%) *	18 (64.3%)	20 (60.6%)	13 (61.9%)	0.957
Have you had problems speaking clearly? *N* (%) *	12 (42.9%)	14 (42.4%)	7 (33.3%)	0.755
M9: problems with teeth crude mean score (SD) Adjusted mean score {SE}	38.1 (34.0) 42.1 {9.8}	30.0 (34.6) 34.9 {11.1}	24.9 (35.6) 24.3 {12.4}	0.402 0.343
Have you had problems with your teeth?, *N* (%) *	16 (57.1%)	17 (51.5%)	10 (47.6%)	0.796
Have you had problems because of losing some teeth?	12 (42.9%)	12 (36.4%)	6 (28.6%)	0.590
Have you had problems chewing?, *N* (%) *	18 (64.3%)	15 (45.5%)	8 (38.1%)	0.153
M10: anxiety crude mean score (SD) Adjusted mean score {SE}	72.0 (31.8) 71.5 {9.3}	51.0 (33.6) 49.6 {10.5}	56.3 (32.3) 50.2 {11.8}	0.043 a 0.004
Have you worried about the results of examination and tests? *N* (%) *	27 (96.4%)	27 (81.8%)	20 (95.2%)	0.107
Have you worried about your health in the future? *N* (%) *	27 (96.4%)	30 (90.9%)	19 (90.5%)	0.643
M11: shoulder problems crude mean score (SD) Adjusted mean score {SE}	34.5 (37.1) 32.6 {8.0}	10.6 (19.0) 7.1 {9.1}	27.8 (32.2) 19.7 {10.1}	0.007 a 0.002 a
Have you had problems raising your arm or moving it sideways? *N* (%) *	12 (42.9%)	6 (18.2%)	9 (42.9%)	0.066
Have you had pain in your shoulder? *N* (%) *	16 (57.1%)	9 (27.3%)	11 (52.4%)	0.043
M12: skin problems crude mean score (SD) Adjusted mean score {SE}	18.7 (24.9) 18.6 {5.6}	10.8 (14.8) 7.3 {6.3}	18.5 (21.8) 11.3 {7.0}	0.245 0.115
Have you had skin problems (e.g., itching, dry)?, *N* (%) *	12 (42.9%)	13 (39.4%)	9 (42.9%)	0.952
Have you had a rash?, *N* (%) *	3 (10.7%)	0 (0.0%)	3 (14.3%)	0.101
Has your skin changed color?, *N* (%) *	11 (39.3%)	7 (21.2%)	10 (47.6%)	0.107
Single-item symptom				
S1: coughing crude mean score (SD) Adjusted mean score {SE}	20.2 (31.9) 23.4 {8.9}	22.2 (35.0) 22.2 {10.1}	15.9 (25.0) 12.5 {11.2}	0.771 0.176
Have you had problems with coughing? *N* (%) *	10 (35.7%)	11 (33.3%)	7 (33.3%)	0.977
S2: opening mouth crude mean score (SD) Adjusted mean score {SE}	38.1 (46.0) 31.2 {10.6}	25.3 (34.4) 13.8 {12.0}	28.6 (39.8) 15.7 {13.4}	0.448 0.301
Have you had problems opening your mouth wide?, *N* (%)*	13 (46.4%)	15 (45.5%)	9 (42.9%)	0.968
S3: social contact crude mean score (SD) Adjusted mean score {SE}	24.0 (41.4) 24.2 {11.2}	18.3 (35.3) 18.3 {12.3}	29.4 (42.3) 29.4 {14.0}	0.632 0.513
Have you had problems going out in public?, *N* (%) *	7 (28.0%)	7 (22.6%)	6 (35.3%)	0.638
S4: neurological problems crude mean score (SD) Adjusted mean score {SE}	45.2 (43.7) 52.9 {9.6}	19.2 (31.2) 27.4 {10.8}	50.8 (34.3) 48.3 {12.1}	0.004 a c 0.005 a
Have you had tingling or numbness in your hands or feet?, *N* (%)	17 (60.7%)	10 (30.3%)	16 (76.2%)	0.002
S5: swelling in the neck crude mean score (SD) Adjusted mean score {SE}	21.4 (34.2) 24.4 {7.0}	6.1 (15.5) 6.7 {7.9}	11.1 (24.3) 7.0 {8.9}	0.066 0.003 a
Have you had swelling in your neck?, *N* (%) *	10 (35.7%)	5 (15.2%)	4 (19.0%)	0.145
S6: weight loss crude mean score (SD) Adjusted mean score {SE}	22.6 (35.2) 25.2 {5.7}	1.0 (5.8) 4.6 {6.5}	4.8 (15.9) 1.4 {7.2}	0.001 a < 0.001 a b
Have you worried that your weight is too low?, *N* (%) *	9 (32.1%)	1 (3.0%)	2 (9.5%)	0.004
S7: problems with wound healing crude mean score (SD) Adjusted mean score {SE}	2.4 (12.6) 1.4 {3.1}	1.0 (12.8) 3.6 {3.5}	0.0 (0.0) 1.8 {3.9}	0.603 0.630
Have you had problems with wound healing?, *N* (%) *	1 (3.6%)	2 (6.1%)	0 (0.0%)	0.512

*SE* standard error, *SD* standard deviation

*Number of patients reporting all response options except for “not at all” and percentages in parenthesis

Post hoc comparisons with *p* < 0.05. **a**Between 1- and 5-year post-treatment. **b**Between 1- and 10-year post-treatment. **c**Between 5- and 10-year post-treatment

### Quality of life assessed with the Orthognathic-QLQ

**Fig. 3:**
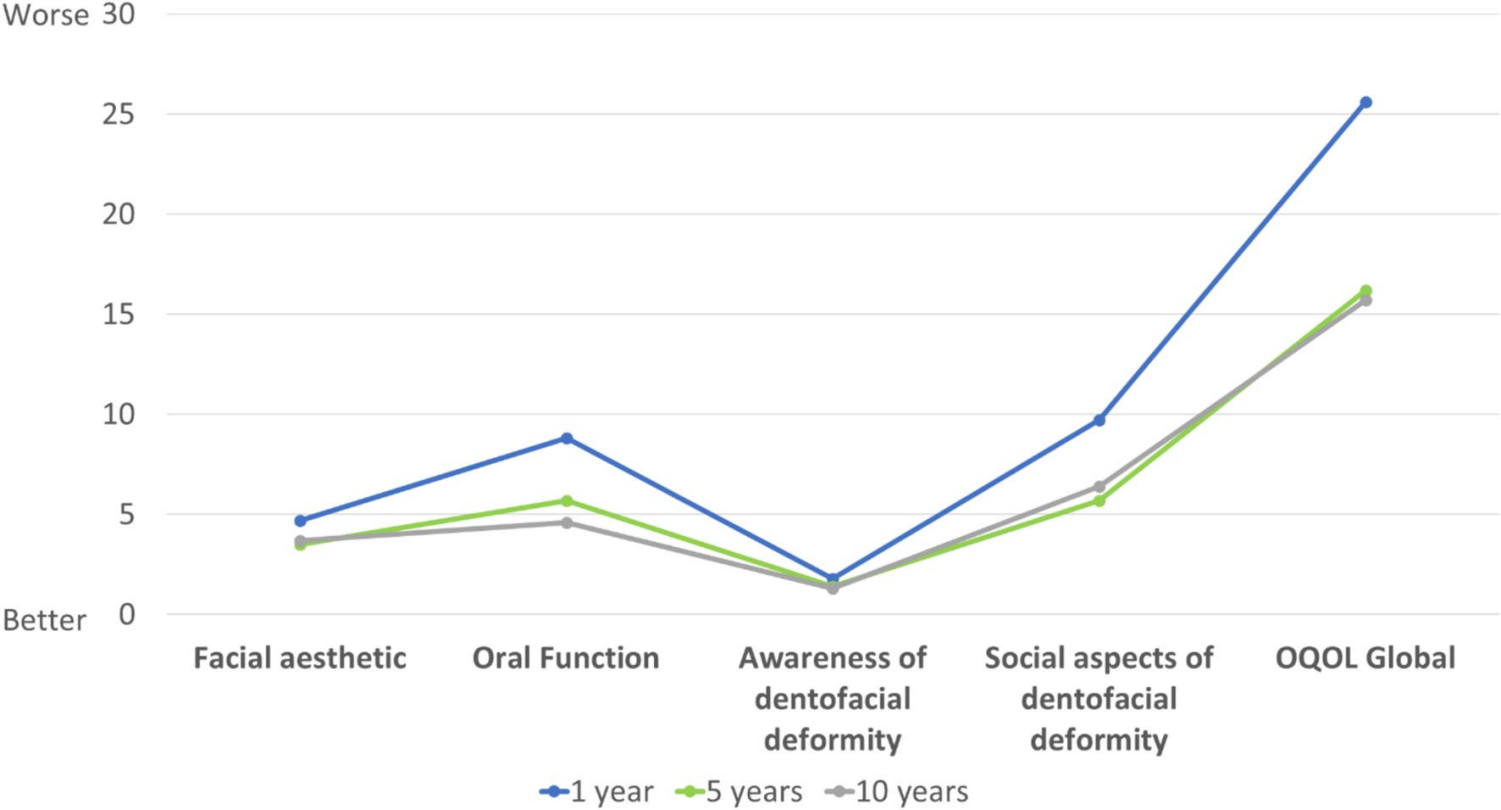
Orthognathic-QLQ questionnaire scores (higher scores indicate worse Oral HRQoL): adjusted means in patients evaluated after 1, 5, and 10 years of completing treatment for head and neck cancer

Oral HRQoL, measured by crude mean scores on the Orthognathic-QLQ, was worse on all QLQ domains in the 1-year group than in the 5- and 10-year groups (Fig. 3). However, when adjusted mean scores were used, these differences were no longer statistically significant. No significant differences were observed between the 5- and 10-year groups on the Orthognathic-QLQ.

Table 4 shows the mean scores on the Orthognathic-QLQ by group. Significant differences were observed in the crude mean global scores (24.6, 11.2, and 11.5 at 1-, 5-, and 10-years, respectively; *p* = 0.026). Significant differences were also found in the crude mean scores of the social aspects of the dentofacial deformity domain (10.0, 3.8, and 4.4, respectively; *p* = 0.018). However, the differences on these two scores disappeared after adjusting for age, sex, tumor and nodal stage (*p* = 0.357 and *p* = 0.296, respectively).

**Table 4:** Orthognathic-QLQ Symptom Index scores after 1, 5, and 10 years of treatment

Variables	Time after completing oncological treatment			p value
	1 year (n = 28)	5 years (n = 33)	10 years (n = 21)	
OQLQ global crude mean score (SD) Adjusted mean score {SE}	24.6 (25.3) 25.6 {5.1}	11.2 (16.3) 16.2 {5.8}	11.5 (19.9) 15.7 {6.5}	0.026 0.357
OQLQ facial esthetic crude mean score (SD) Adjusted mean score {SE}	4.4 (5.7) 4.7 {1.2}	1.8 (3.8) 3.5 {1.3}	1.8 (4.6) 3.7 {1.5}	0.068 0.183
Self-conscious about appearance of my teeth, *N* (%) *	8 (28.6%)	8 (24.2%)	3 (14.3%)	0.494
Don’t like seeing side view of face (profile), *N* (%) *	8 (28.6%)	3 (9.1%)	1 (4.8%)	0.033
Dislike having photograph taken, *N* (%) *	9 (32.1%)	4 (12.1%)	3 (14.3%)	0.113
Dislike being seen on video, *N* (%) *	10 (35.7%)	6 (18.2%)	3 (14.3%)	0.145
Self-conscious about appearance, *N* (%) *	8 (28.6%)	2 (6.1%)	1 (4.8%)	0.015
OQLQ oral function crude mean score (SD) Adjusted mean score {SE}	8.4 (7.5) 8.8 {1.8}	4.6 (5.8) 5.7 {2.1}	4.7 (6.6) 4.6 {2.3}	0.058 0.240
Problems biting, *N* (%) *	16 (57.1%)	14 (42.4%)	8 (38.1%)	0.351
Problems chewing, *N* (%) *	18 (64.3%)	15 (45.5%)	8 (38.1%)	0.153
Avoid eating some foods, *N* (%) *	16 (57.1%)	14 (42.4%)	6 (28.6%)	0.134
Do not like eating in public, *N* (%) *	12 (42.9%)	10 (30.3%)	7 (33.3%)	0.578
Pain in face/jaw, *N* (%) *	13 (46.4%)	5 (15.2%)	5 (23.8%)	0.022
OQLQ awareness of dentofacial deformity Crude mean score (SD) Adjusted mean score {SE}	1.8 (3.3) 1.8 {0.7}	0.9 (2.7) 1.4 {0.8}	0.6 (2.2) 1.3 {0.9}	0.287 0.867
Spend time studying face, *N* (%) *	6 (21.4%)	5 (15.2%)	1 (4.8%)	0.262
Spend time studying teeth, *N* (%) *	8 (28.6%)	5 (15.2%)	2 (9.5%)	0.194
Stare at people’s teeth, *N* (%) *	3 (10.7%)	3 (9.1%)	1 (4.8%)	0.753
Stare at people’s faces, *N* (%) *	4 (14.3%)	4 (12.1%)	1 (4.8%)	0.552
OQLQ social aspects of dentofacial deformity Crude mean score (SD) Adjusted mean score {SE}	10.0 (11.0) 10.2 {2.1}	3.8 (6.7) 5.6 {2.4}	4.4 (8.7) 6.1 {2.7}	0.018 a 0.296
Cover mouth when meeting people, *N* (%) *	18 (64.3%)	5 (15.2%)	2 (9.5%)	< 0.001
Worry about meeting people, *N* (%) *	19 (67.9%)	6 (18.1%)	3 (14.3%)	< 0.001
Worry people will make hurtful comment, *N* (%) *	15 (57.7%)	14 (42.4%)	5 (25.0%)	0.085
Lack confidence socially, *N* (%) *	11 (39.3%)	11 (33.3%)	5 (23.8%)	0.521
Do not like smiling, *N* (%) *	8 (28.6%)	3 (9.1%)	2 (9.5%)	0.076
Get depressed about appearance, *N* (%) *	13 (46.4%)	6 (18.2%)	4 (19.0%)	0.028
Sometimes think People are starring, *N* (%) *	8 (28.6%)	5 (15.2%)	3 (14.3%)	0.328
Comments about appearance upset me, *N* (%) *	12 (44.4%)	10 (30.3%)	5 (23.8%)	0.287

*SE* standard error, *SD* standard deviation

*Number of patients reporting all response options except for “not at all” and percentages in parenthesis

Post-hoc comparisons with *p* < 0.05. **a** Between 1- and 5-year post-treatment, **b** Between 1- and 10-year post-treatment, **c** Between 5- and 10-year post-treatment

### Feasibility of telephone interviews

All three questionnaires were administered by a professional interviewer. Administration was straightforward and practical. All participants were able to complete the questionnaires without difficulty, and none of the patients had to be excluded due to difficulties in completing the questionnaires.

## Discussion

The main aim of this study was to assess oral HRQoL at 1, 5, and 10 years in a cohort of patients treated for H&N cancer. This study shows that H&N cancer had a greater negative impact on HRQoL at 1 year than at 5 and 10 years. No significant between-group differences were observed in crude or adjusted mean scores on the FACT H&N. Similarly, no significant between-group differences were observed in the adjusted mean scores on the Orthognathic-QLQ. However, we did detect significant differences between the 1-year group and the 5- and 10-year groups on five of the EORTC QLQ-H&N43 multi-item scales and on three single-item scales. Overall, the combination of the EORTC QLQ-H&N43 and the Orthognathic-QLQ appeared to be more useful for assessing general and oral HRQoL.

The only instrument that detected significant differences in HRQoL between the 1-year and 5- or 10-year groups was the EORTC QLQ-H&N43. This instrument identified significant differences on five multi-item scales (mouth pain; senses; body image; anxiety; and shoulder problems) and three single-item scales (neurological problems, neck swelling, and weight loss), indicating a greater negative impact on QoL on those domains at 1-year post-treatment. This finding suggest that this questionnaire may be more valuable for assessing general and oral HRQoL than the FACT H&N, which was not capable of detecting any differences between the three time points. Although the Orthognathic-QLQ did not detect any significant differences between groups, we believe this instrument provides useful data that is complementary to more general instruments such as the EORTC QLQ-H&N43. In this regard, it is important to point out that the Orthognathic-QLQ does not directly measure the three most common sequalae of H&N treatment (trismus, xerostomia and dysphagia). Although this instrument can be used to indirectly assess some of the consequences of these adverse effects, the true value and purpose of the Orthognathic-QLQ is to assess the need for dental care, which is why combining it with the EORTC QLQ-H&N43 provides a more comprehensive vision of global HRQoL, including dental needs.

H&N cancer can have a significant negative impact on oral health. Treatment (surgery, radiation therapy, and chemotherapy) also plays an important role in adverse oral health effects, including mucositis, xerostomia, hypogeusia, dental caries, and osteoradionecrosis. These complications impair vital bodily functions such as eating, speaking, and swallowing, thus reducing HRQoL [11, 12]. As survival rates for H&N cancer have increased in recent years, QoL has become a significant concern for survivors and HRQoL-related outcomes have become increasingly important. The oral and dental side effects from treatment include dry mouth and sticky saliva and treatment can also disrupt crucial functions such as chewing and swallowing, which in turn can affect the patient’s nutritional intake and esthetics, potentially leading to isolation and further reducing HRQoL [13, 14]. The psychological impact of dental issues, which include self-consciousness about oral appearance and fear of social stigma, can also negatively affect patient well-being.

In this series of patients, we observed a clear pattern—evident on all three HRQoL instruments—of worse HRQoL at 1-year post-treatment than at 5 or 10 years. However, we only observed statistically significant differences between groups (adjusted means) on one of the instruments (EORTC QLQ-H&N43), and mainly only between the 1- and 5-year groups. The only significant differences between the 1- and 10-year groups on the EORTC QLQ-H&N43 were on the senses (M3) and weight loss (S6) domains. There were no significant differences between the 5- and 10-year groups on any of the scales. These findings seem to indicate that H&N cancer and treatment induces an important decline in HRQoL in the 12-month period following treatment completion, but that HRQoL then improves or stabilizes over the long-term.

Several treatments are available to alleviate discomfort from xerostomia and mucositis, including topical analgesics, saliva substitutes, and oral moisturizers. These treatments help patients to maintain oral function and nutritional intake. In our series, dry mouth/sticky saliva and swallowing were significantly worse (EORTC QLQ-H&N43) in the 1-year group than in the 5- and 10-year groups, suggesting that these two symptoms improve over time. Several studies have shown that H&N cancer patients, particularly those with oral cavity cancer, should receive routine dental care after treatment to prevent, treat, or minimize complications affecting dental health [15, 16]. Orthodontics and restorative dentistry are often essential in this patient population [17].

Although we did not observe any cases of osteoradionecrosis in this series, this condition is a potential complication of tooth extraction in irradiated bone, typically affecting the mandible [18]. However, prior to initiating radiotherapy, it is advisable to check for teeth that may require extraction, such as partially erupted teeth with retained root tips, or with periodontal involvement. Close communication among the medical team (medical and radiation oncologists, surgeons, and dentists) is crucial to ensure appropriate assessment. A complete oral and dental examination is recommended before initiating any oncological treatment.

Oral mucositis is a common side effect of cancer therapy and dental treatment is not feasible when patients are undergoing radiotherapy and/or chemotherapy [19]. Jawad et al. reviewed dental treatment in H&N cancer patients before, during, and after radiotherapy [20].

There were significant differences in adjusted mean scores between the 1- and 5-year groups (all worse in the 1-year group) on numerous multi-items on the EORTC QLQ-H&N43 including pain in the mouth, problems with senses, body image, anxiety, shoulder problems, neurological problems, swelling in the neck, weight loss, and sexuality. By contrast, significant differences between the 1-year and 10-year groups were only observed on three items (senses, anxiety and sexuality). This finding suggests that most—but not all—of the adverse effects of treatment tend to disappear over time.

Taste loss is another common symptom in patients treated with radiotherapy. A previous study carried out by our group showed that radiotherapy-induced taste dysfunction decreases the perception of sweet, bitter, salty, sour, and umami tastes during the first 6 weeks of treatment, with symptom recovery occurring 1 year after initiation of radiotherapy [21].

The most frequently reported symptoms on the FACT H&N Symptom Index in the three groups were 1) concerns that the condition will worsen (> 90% of respondents in all three groups) and 2) the presence of pain (> 75% in the 1- and 5-year groups and 95% in the 10-year group). Consistent with these results, the most commonly reported problems (approximately 90% of respondents in all three groups) on the EORTC QLQ-H&N43 were 1) worries about test results and 2) worries about future health status. Compared to the 5- and 10-year groups, a significantly higher proportion of patients in the 1-year group reported “pain or soreness in mouth” and “pain in jaw” (46% and 64%, respectively vs. < 35% at 5 and 10 years).

Close monitoring of the oral HRQoL of H&N cancer patients could help with the early identification of emerging issues, which could, in turn, facilitate selection of the most suitable strategy, such as rehabilitation to restore oral function and esthetics. Prosthodontic interventions (e.g., removable prostheses and maxillofacial prostheses) may be necessary in some cases to restore oral function, speech intelligibility, and improve facial esthetics, thereby improving psychosocial well-being and QoL. Speech therapy and swallowing rehabilitation programs can address functional impairments. In our series, there were significant differences between the three groups in terms of the patients’ reported ability to swallow naturally and easily (FACT H&N Symptom Index). Only 60.7% of patients in the 1-year group indicated that they could swallow without difficulties versus 69.7% and 85.7% in the 5- and 10-year groups. In patients with dysphagia, studies show that multidisciplinary collaboration among radiation oncologists, medical oncologists, and dentists is essential to improve this condition, and thus QoL [17].

In a previously published pilot study, our group administered the FACT H&N Symptom Index and the EORTC QLQ-H&N43 to evaluate the impact of radiotherapy on QoL [22]. In line with the present study, QoL was significantly better in patients at 5-years post-treatment than at 1 year. That study also revealed important limitations in both of those instruments especially the FACT H&N Symptom Index—with regard to their ability to detect problems that could be alleviated with dental treatment. In this regard, the findings of that pilot study underscore the need for instruments specifically developed to assess dental HRQoL rather than just cancer-specific HRQoL. In fact, the present study was carried out to overcome the limitations of that pilot study by including a specific oral HRQoL instrument (Orthognathic-QLQ), a larger sample size, and longer follow-up [22]. Notwithstanding the differences between the two studies, the findings of the present study are largely consistent with those obtained in the pilot study, confirming that H&N cancer has a greater negative impact on QoL at 1 year than at 5 or 10 years.

The FACT H&N Symptom Index contains only three items to assess oral health and function (swallowing, and mouth, throat or neck pain; and ability to eat solid foods). By contrast, the EORTC QLQ-H&N43 includes five multi-item scales related to oral QoL (dry mouth/sticky saliva, pain in the mouth, social eating, swallowing, and problems with teeth) and two single-item symptoms (opening mouth and problems with wound healing). In this regard, the EORTC instrument could be of particular interest to orthodontists. The Orthognathic-QLQ covers four domains: facial esthetics, oral function, awareness of dental deformity, and overall dental quality of life.

The telephone administration of all three study questionnaires by an experienced interviewer was simple and feasible. This finding is consistent with previous reports which have found that telephone administration of HRQoL questionnaires (including the EORTC QLQ-C30 for cancer patients) is equivalent to patient-completed administration [23, 24].

In the present study, we evaluated three different questionnaires designed to assess oral function and HRQoL. However, it should be noted that numerous other tools are available. Although a detailed comparison of those tools is beyond the scope of the current study, a recently published review (2023) by In't Veld et al. provides a comprehensive overview of the available tools, including an evaluation of 16 different questionnaires designed to assess in patients with H&N cancer [25].

### Study limitations

This study has several limitations, including the relatively limited sample size (n = 82). In addition, for purposes of this study, we assumed that all H&N tumors, regardless of their anatomic localization, affect patients equally to compare the questionnaires. In addition, given that the primary interest was to assess the need for dental care, surgical patients were not included.

## Conclusion

The findings of this study show that H&N cancer has a greater negative impact on HRQoL—measured by the FACT H&N Symptom Index, the EORTC QLQ-H&N43 and the Orthognathic-QLQ—at 1-year post-treatment than at 5 and 10 years. Telephone administration of these questionnaires by a trained interviewer is straightforward and feasible. The combination of the EORTC QLQ-H&N43 and the Orthognathic-QLQ appears to be more useful than the FACT H&N Symptom Index for assessing HRQoL, particularly oral and dental HRQoL.

Patients with head and neck cancer face numerous disease- and treatment-related challenges, including oral and dental health. Given the potential for severe treatment-related complications in these patients, it is essential to assess oral health and the need for dental care to prevent or ameliorate the negative impact of treatment on HRQoL. The findings of the present study underscore the importance of assessing oral health and providing dental care to restore oral function and esthetics. Through the application of preventive measures, supportive care practices, and rehabilitation approaches, healthcare providers can lessen the negative impact of cancer treatment on oral health and boost the overall well-being of H&N cancer survivors.

## Data Availability

Not applicable.
